# The benzodiazepine-like natural product tilivalline is produced by the entomopathogenic bacterium *Xenorhabdus eapokensis*

**DOI:** 10.1371/journal.pone.0194297

**Published:** 2018-03-29

**Authors:** Hendrik Wolff, Helge B. Bode

**Affiliations:** 1 Fachbereich Biowissenschaften, Merck Stiftungsprofessur für Molekulare Biotechnologie, Goethe-Universität Frankfurt, Frankfurt am Main, Germany; 2 Buchmann Institute for Molecular Life Sciences (BMLS), Goethe-Universität Frankfurt, Frankfurt am Main, Germany; Wageningen Universiteit, NETHERLANDS

## Abstract

The pyrrolobenzodiazepine tilivalline (**1**) was originally identified in the human gut pathobiont *Klebsiella oxytoca*, the causative agent of antibiotic-associated hemorrhagic colitis. Here we show the identification of tilivalline and analogs thereof in the entomopathogenic bacterium *Xenorhabdus eapokensis* as well as the identification of its biosynthesis gene cluster encoding a bimodular non-ribosomal peptide synthetase. Heterologous expression of both genes in *E*. *coli* resulted in the production of **1** and from mutasynthesis and precursor directed biosynthesis 11 new tilivalline analogs were identified in *X*. *eapokensis*. These results allowed the prediction of the tilivalline biosynthesis being similar to that in *K*. *oxytoca*.

## Introduction

Entomopathogenic gammaproteobacteria of the genus *Xenorhabdus* live in symbiosis with nematodes of the genus *Steinernema*. Soil living infective juvenile nematodes are carrying *Xenorhabdus*, hunting and killing insect larvae as food source and reproductive space [1–3]. To maintain this specific lifestyle (bypass of the insect immune system, killing and lysis of the insect, defending the insect corpse from microorganisms and other food competitors) *Xenorhabdus* produce a wide variety of natural products [4–7]. If insect cadaver depletion occurs, a new generation of juvenile nematodes and bacteria re-associate and emerge from the cadaver in search of a new insect prey [2].

Most natural products (NPs) of *Xenorhabdus* are produced by non-ribosomal peptide synthetases (NRPSs), polyketide synthases or hybrids thereof. The NRPS module architecture can often be identified using *in silico* methods such as antiSMASH [8] that predicts enzyme domain function and also shows the biosynthetic gene cluster (BGC) similarity to other BGCs with known natural products. In NRPS-based biosynthesis several catalytically active domains can be grouped into modules that are responsible for the activation and processing of the individual building blocks usually amino acids. The adenylation (A) domain specifically selects an amino acid substrate and activates it in an ATP dependent manner to attach it covalently to the downstream thiolation or peptidyl carrier protein domain (T). The condensation (C) domain joins adjacent T domain bound amino acids and a growing peptide chain is transferred downstream. A NRPS starting module typically consists only of an A domain and a T domain whereas the final termination module often includes a thioesterase (TE) or a reductase (Re) domain to release the peptide from the NRPS enzyme complex [9].

Besides accelerating developments in genome analysis, recent developments in ultra-performance liquid chromatography (UPLC) coupled to high resolution mass spectrometry (HR-MS) have allowed high-throughput chemical analysis of microbial cultivation samples. Subsequent data mining techniques like network analyses [10] are used to extract bacterial NP profiles which in turn can be integrated in genome mining approaches to achieve a broad NP producer characterization and help to identify new compounds [11].

Pyrrolobenzodiazepines (PBDs) are a class of natural products known for their sequence specific DNA binding ability, which causes cytotoxicity [12]. Bacteria occupying different ecological niches are reported to produce PBDs [13]. For example, terrestrial actinobacteria such as *Streptosporangium sibiricum*, *Streptomyces refuineus* sbsp. *thermotolerans* or *Streptomyces achromogenes* produce sibiromycin [14,15], anthramycin [16–18] and tomaymycin [19–21], respectively. The PBD tilivalline (TV, **1**), is produced by *Klebsiella oxytoca* [22–24], a human gut pathobiont [25,26]. *Klebsiella oxytoca* shifts from an anti-inflammatory commensal to a pro-inflammatory pathobiont during antibiotic treatment [27,28]. When antibiotic driven enterobacterial overgrowth occurs and **1** is produced in higher amounts by *Klebsiella oxytoca*, activation of mucosal immune cells leads to a damage of the intestinal epithelium [24,27]. This in turn increases intestinal permeability and chronic activation of the immune system resulting the pathology of antibiotic associated hemorrhagic colitis (AAHC) [28–31].

Here, the identification of the TV BGC in several entomopathogenic *Xenorhabdus* strains and production of **1** by *X*. *eapokensis* strain DL20 [32] was shown under laboratory conditions, as previously described briefly [33]. Additionally, a precursor directed biosynthesis approach led to increased production of TV and analogs thereof. Furthermore, heterologous expression of the TV BGC of *X*. *indica* in *E*. *coli* resulted in TV production and a mutasynthesis approach resulted in production of several TV analogs highlighting the enzyme promiscuity.

## Results

### Tilivalline gene cluster analysis

**Fig 1:**
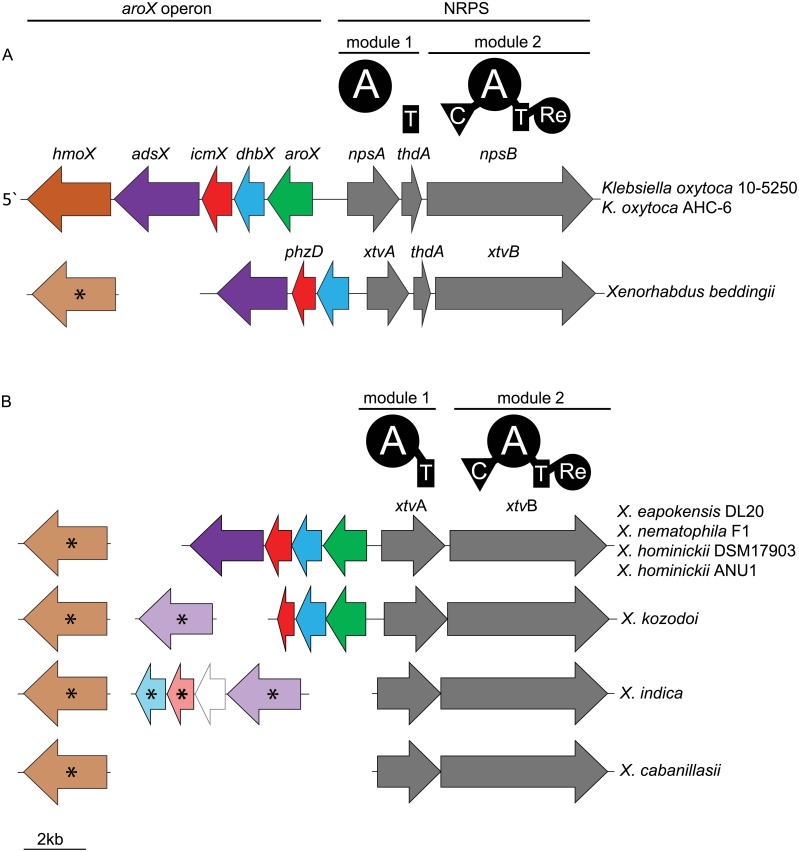
Tilivallin (1) biosynthetic gene cluster (BGC) composition in (A) *Klebsiella oxytoca* sp. and *Xenorhabdus beddingii* and (B) additional *Xenorhabdus* strains. The TV BGC contains the *aroX* operon and encodes a bimodular NRPS (grey arrows; in *Klebsiella* sp. NpsAB and ThdA, in *Xenorhabdus* sp. XtvAB) with corresponding domain architecture annotation according to antiSMASH analysis and [28]). Note the differences in NRPS architecture in (A, freestanding) and (B, fused A-T didomain). Abbreviations: A (adenylation), T (thiolation), C (condensation), Re (reductase) domain. Colored arrows show genes of the *aroX* operon encoding the following enzymes: Orange (HmoX, 4-hydroxyphenyl acetate-3-monoxygenase), violet (AdsX, 2-amino-2-deoxy-isochorismate synthase), red (IcmX or PhzD homolog, respectively, isochorismatase), blue (DhbX, 2,3-dihydro-dehydrogenase), green (AroX, 2-keto-3-deoxy-D-arabinoheptulosonate phosphate synthase), white (2,3-dihydroxybenzoate-AMP ligase of chrysobactin BGC). Arrows with shaded colors and asterisk indicate that corresponding genes are not encoded in TV BGC but elsewhere in the genome.

Genomic mining of 26 *Xenorhabdus* genomes (S1 Table) using antiSMASH 3.0 [8] revealed the presence of a BGC encoding a NRPS in *X*. *indica*, *X*. *eapokensis* strain DL20, *X*. *hominickii* DSM17908 and strain ANU1, *X*. *kozodoi*, *X*. *cabanillasii*, *X*. *nematophila* F1 and *X*. *beddingii* with an average amino acid identity >50% to the TV NRPS of *Klebsiella oxytoca* [28] (Fig 1, S2 Table). In *K*. *oxytoca* the TV BGC consists of five genes described as the *aroX* operon and three genes (6.4 kbp in total size) encoding the A domain NpsA, the T domain ThdA and the NRPS modul NpsB (consisting of a C, an A, a T and a Re domain) [28]. Notably, NpsA and ThdA are freestanding domains in *K*. *oxytoca* and *X*. *beddingii* (Fig 1). In all other *Xenorhabdus* genomes the NRPS is encoded on two genes, hereafter named *xtvAB* (from *X**enorhabdus* tilivalline) where in contrast to *Klebsiella* and *X*. *beddingii xtvA* encodes a A-T di-domain as starting module.

The *aroX* operon of *K*. *oxytoca* contains five genes (*hmoX*, *adsX*, *icmX*, *dhbX*, *aroX*) coding for enzymes which are thought to be responsible for synthesis of the TV precursor 3-hydroxy anthranilic acid [28,34] (Fig 1, S2 Table). In general, none of the analyzed *Xenorhabdus* sp. genomes harbor the full *aroX* operon as described for *K*. *oxytoca* because the anthranilate-3-monoxygenase HmoX homolog is not encoded in the TV BGC but elsewhere on the genome. *X*. *eapokensis* strain DL20, *X*. *nematophila* F1, and *X*. *hominickii* spp. encode several *aroX* operon genes as part of the TV BGC but *X*. *beddingii* is lacking *aroX* (encoding 2-keto-3-deoxy-D-arabinoheptulosonate phosphate synthase) and *X*. *kozodoi* encodes the 2-amino-4-deoxychorismate synthase AdsX somewhere else in the genome. Interestingly, *X*. *indica* encodes AdsX, the IcmX homolog PhzD and the 2,3-dihydro-2,3-dihydroxybenzoate dehydrogenase DhbX not as part of the TV BGC, but as part of a predicted chrysobactin siderophore BGC [35]. Finally, *X*. *cabanillasii* is lacking the aforementioned four *aroX* operon genes and no homologs could be identified in the referred genomic data (Fig 1, S2 Table).

### Production of tilivalline and natural analogs in *X*. *eapokensis*

In order to investigate the tilivalline production, six *Xenorhabdus* strains harboring the TV BGC were chosen for cultivation and mass spectrometric analysis. The strains were grown in either LB broth, SF-900 or Schneider′s insect medium including Amberlite XAD-16, which was harvested after three days of incubation and extracted with butanol. Subsequently, these extracts were analyzed using UPLC-HR-MS/MS, to test whether **1** is produced under laboratory conditions (S1 Table).

**Fig 2:**
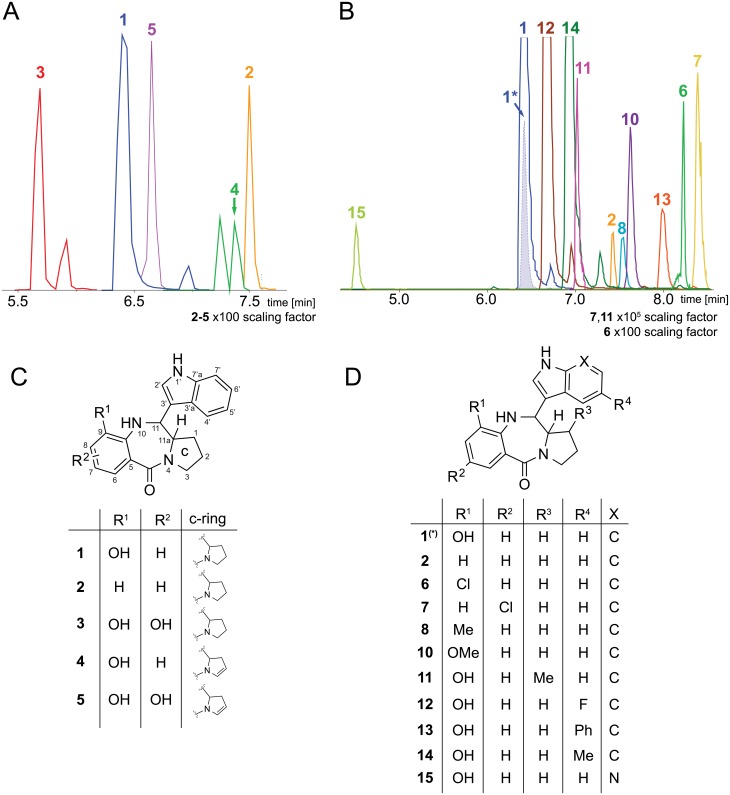
UPLC-HRMS/MS analysis of crude extracts of *X*. *eapokensis* grown in SF-900 medium. (A) Extracted ion chromatograms (EIC) of natural produced TV (**1**), and derivatives thereof (**2**–**5**) with structures according to (C). (B) EICs of **1** and derivatives thereof (**2, 6**–**8, 10**–**15**) with structures according to (D) of precursor directed feeding experiment (details see text). Note: 1* shows TV signal intensity in an extract without supplementation of 3-hydroxy anthranilic acid.

Only the *X*. *eapokensis* extract exhibited a molecular feature of *m/z* 334.1550 [M+H]^+^ (Fig 2, S1 Fig) with a predicted sum formula of C_20_H_19_N_3_O_2_ (Table 1) characteristic for TV (**1**) [23]. A subsequent analysis of the MS fragmentation data of this molecular feature to reference data of **1** produced by *K*. *oxytoca* [29] and a synthetic TV standard (S1 Fig) provided additional evidence that the molecule is indeed **1** produced by *X*. *eapokensis*. Quantification showed that **1** is produced in a range of in 0.032 mg/L in LB broth, 0.41 mg/L in Schneider′s insect medium and 2.06 mg/L in SF-900 insect medium.

**Table 1:** Overview of TV, derivatives thereof and putative biosynthetic precursors described in this work.

compound	name	sum formula [M+H]^+^	expected [M+H]^+^	measured [M+H]^+^	Δppm
**1**	Tilivallin (TV)	C_20_H_20_N_3_O_2_	334.1550	334.1550	0
**2**	9-deoxy-TV[36]	C_20_H_20_N_3_O	318.1601	318.1597	1.5
**3**	dihydroxy-TV	C_20_H_20_N_3_O_3_	350.1499	350.1497	1.4
**4**	dehydro-TV	C_20_H_18_N_3_O_2_	332.1394	332.1411	-5.2
**5**	dehydro-dihydroxy-TV	C_20_H_18_N_3_O_3_	348.1343	348.1337	0.6
**6**	9-chloro PBD	C_20_H_19_N_3_OCl	352.1211	352.1214	-0.7
**7**	7-chloro PBD	C_20_H_19_N_3_OCl	352.1211	352.1211	0
**8**	9-methyl PBD	C_21_H_22_N_3_O	332.1757	332.1755	0.8
**9**	7-methyl PBD	C_21_H_22_N_3_O	332.1757	332.1757	0
**10**	9-methoxy PBD	C_21_H_22_N_3_O_2_	348.1707	348.1708	-0.3
**11**	1-methyl-9-hydroxy PBD	C_21_H_22_N_3_O_2_	348.1712	348.1706	0.4
**12**	9-hydroxy-5’-fluoro PBD	C_20_H_19_N_3_O_2_F	352.1461	352.1451	0.5
**13**	9-hydroxy-5’-phenyl PBD	C_26_H_24_N_3_O_2_	410.1863	410.1863	0
**14**	9-hydroxy-5’-methyl PBD	C_21_H_22_N_3_O_2_	348.1712	348.1706	0.2
**15**	9-hydroxy-azaindole PBD	C_19_H_19_N_4_O_2_	335.1508	335.1498	1.3
**16**	Tilimycin[36]/Kleboxymycin[34]	C_12_H_15_N_2_O_3_	235.1077	235.1076	0.6
**17**	TV keto precursor	C_12_H_13_N_2_O_3_	233.0921	233.0920	0.2

**Fig 3:**
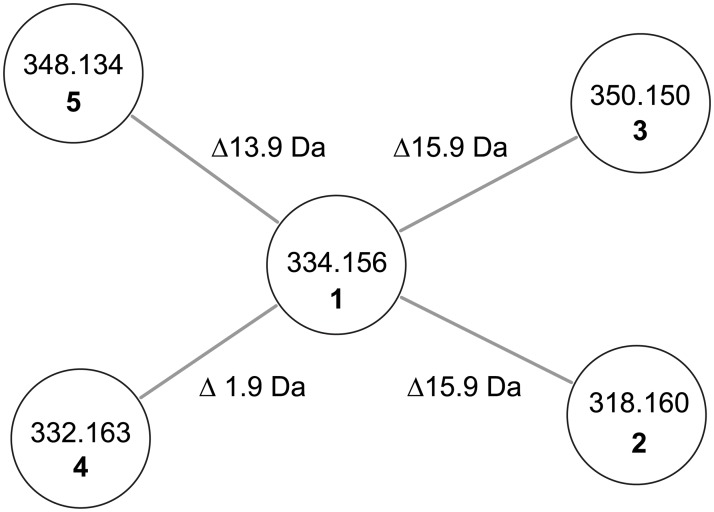
TV specific subnetwork analysis of a *X*. *eapokensis* cultivation in SF-900 insect medium. Nodes (circles) are labeled with precursor ion masses [M+H]^+^ of TV (**1**) and derivatives thereof (**2**–**5**). Precursor mass differences are depicted on connecting edges (grey lines).

**Fig 4:**
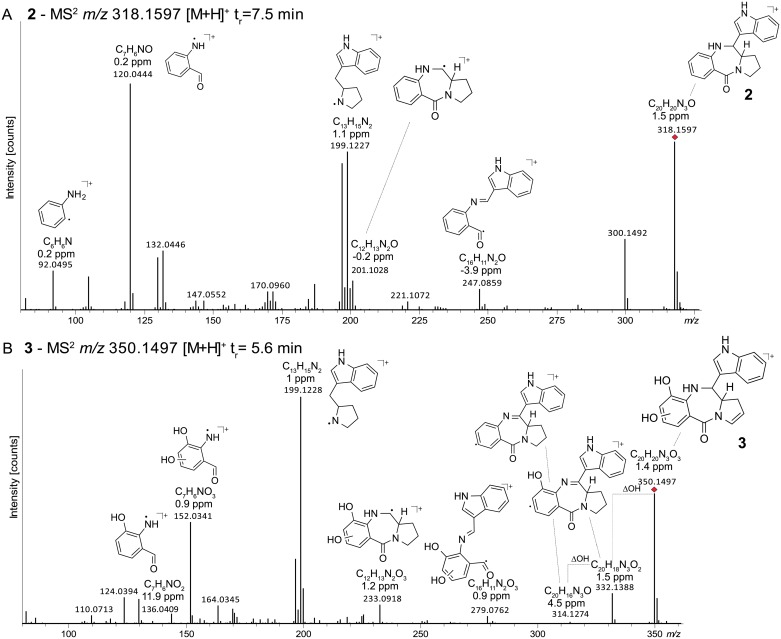
UPLC-HRMS/MS analysis of a crude extract of *X*. *eapokensis* grown in SF-900 medium. (A) MS/MS spectrum of 9-deoxy-TV (**2**). (B) MS/MS spectrum of dihydroxy-TV (**3**). Signals are annotated with predicted sum formulas, detection errors and putative molecule fragment structures. Red diamonds indicate precursor ions.

A subsequent MS/MS analysis on *X*. *eapokensis* extraction samples using the GNPS network analysis tool [10] revealed a subnetwork containing TV related compounds (**2**–**5**), (Figs 2 and 3, Table 1) which were subjected to a detailed MS/MS analysis. This allowed the identification of the molecular feature of *m/z* 318.1597 [M+H]^+^ (**2**) as 9- deoxy-TV due to a mass shift of 15.9 Da in the network analysis. Compared to **1** this indicates that the hydroxyl group is missing (Fig 4a) as was also described previously from *K*. *oxytoca* [36]. Its typical MS/MS characteristics (Fig 4a) are two fragment ions namely *m/z* 201.1028 [M+H]^+^ that show a higher signal intensity than an expected second isotopic signal of ubiquitous PBD fragment ion *m/z* 199.1227 [M+H]^+^ would cause, and *m/z* 120.0444 [M+H]^+^, the PBD A-ring fragment without substitutions. Next, the molecular feature of *m/z* 350.1497 [M+H]^+^ (**3**) shows the same 15.9 Da parent mass difference in network analysis as **2** but now as additional mass compared to **1** (Fig 3). Its MS/MS analysis (Fig 4b) shows a characteristic double loss of 18 Da from *m/z* 350.1497 [M+H]^+^ parent ion, most likely attributable to neutral losses of two hydroxyl groups as PBD A-ring substituents. This is also reflected in the occurrence of fragment ions *m/z* 233.0918 [M+H]^+^ and A-ring fragment *m/z* 152.0341 [M+H]^+^, both 15.9 Da larger than the mono hydroxyl substituted fragment ion counterparts *m/z* 217.0972 [M+H]^+^ and *m/z* 136.0385 [M+H]^+^ of **1**. Based on these observations, **3** is determined as dihydroxy TV. In accordance to TV structure data [28], biosynthesis proposals [24,34,36], and precursor directed feeding experiments (see Results below) one A-ring hydroxyl group might be located at position 9 part of the PBD A-ring (Fig 2). The exact position of the second hydroxyl group remains elusive but might be at position 7 or 8 as different potential anthranilate hydroxylases are encoded in the *X*. *eapokensis* genome (see Discussion) (S2 Table). Finally, molecular features of *m/z* 332.1411 [M+H]^+^ (**4**) (S2a Fig) and *m/z* 348.1337 [M+H]^+^ (**5**) (S2b Fig) are characterized as dehydro analogs of **1** and **3** by the mass shift of 1.9 Da of **4** to **1** and **3** to **5**, respectively, as shown in the network analysis (Fig 3). Albeit a distinct assignment of the double bound position is not achievable with the applied methods, characteristic A-ring MS/MS fragment masses *m/z* 197.1073 [M+H]^+^ and *m/z* 215.0811 [M+H]^+^ of **4** and *m/z* 330.1234 [M+H]^+^ of **5**, indicate desaturation in the PBD C-ring (S2 Fig). In summary, *X*. *eapokensis* strain DL20 cultivated under laboratory conditions produces TV (**1**) and four derivatives thereof: 9-deoxy-TV (**2**) and the three hitherto undescribed analogs dihydroxy TV (**3**), dehydro TV (**4**) and dehydro-dihydroxy TV (**5**) (Table 1).

Furthermore, two ions were detected in *X*. *eapokensis* cultivation extract that correlate to the first NRPS derived intermediate named tilimycin or kleboxymycin (**16**) [24,34,36] and its keto analog **17**, respectively (Table 1, S4 Fig). Both are lacking the characteristic indole substituent at the C11 position, instead harboring a hydroxyl group (**16**, carbinolamine precursor), or a keto group (**17**, keto precursor). Mass spectra of **16** (S4 Fig) are in accordance with published data of Tse *et al*. [34] indicating that not all NRPS-produced intermediates are directly converted to **1** via indole addition.

### *X*. *eapokensis* precursor directed biosynthesis and enhanced TV production

A precursor directed biosynthesis approach tested the capability of *X*. *eapokensis* to generate TV and non-natural analogs thereof by supplementing the SF-900 culture broth with different substituted 2-amino benzoic acids (ABA), indoles or prolines (S3 Table), similar to precursor directed biosynthesis approaches described for sibiromycin [37] or tomaymycin [21]. Cultivation extracts were analyzed by UPLC-HRMS/MS (Fig 2). First, comparison of TV production in pure SF-900 medium to SF-900 medium supplemented with either 1 mM 3-hydroxy anthranilic acid (3-HAA), 0.1 mM or 1 mM indole revealed an increase in TV production from 2.06 mg/L in the wildtype to 2.9 mg/L, 3.6 mg/L, and 6.2 mg/L TV, respectively. The largest amount of **1** (6.4 mg/L) was obtained from supplementation with 1 mM indole and 1 mM 3-HAA.

Second, cultures were supplemented with different ABAs, indole or proline derivatives (S3 Table) and extracts were subjected to detailed MS/MS fragmentation analysis. This revealed production of several non-natural TV analogs with different substitutions in the A-ring (**6**–**8**, **10**) and C-ring (**11**) of the PDB, or variations of the indole moiety (**12**–**15**) (S3 Fig, S3 Table). The first set of added precursors tested the *X*. *eapokensis* XtvA A domain substrate flexibility regarding acceptance of halogenated, methylated or methoxylated aminobenzoic acid (ABA) derivatives (S3 Table). The results (Fig 2) suggest a preference of ABAs substituted at the 3-position. Qualitatively, highest product signals are observed for 3-HAA supplementation resulting in **1** followed by preferences of 3-methoxy ABA, 3-methyl ABA, 3-chloro ABA and 5-chloro ABA, resulting in TV analogs **10**, **8**, **6**, **7**, respectively (Fig 2b). Notably, none of the expected TV derivatives were observed in *X*. *eapokensis* after supplementation with 5-methyl ABA or 5-methoxy ABA but production of **1** was as high as in SF-900 medium without supplementation (data not shown). This indicates a substrate preference of XtvA. In a similar manner, the XtvB A domain substrate flexibility was tested by supplementation of different proline analogs, namely 3-methyl, 3-benzyl, and 4-hydroxy proline and pipecolic acid but only incorporation of 3-methyl proline was observed, resulting in TV analog **11** (Fig 2b, S3 Fig). Finally, indole derivatives with substitution at position 5 (5-fluoro-, 5-phenyl-, 5-methyl) and 7-azaindole were incorporated successfully, resulting in TV analogs **12**–**15** (Fig 2b, S3 Fig). In summary, *X*. *eapokensis* produces high amounts of **1** in the presence of high concentrations of 3-HAA acid and indole. If the bacteria are additionally supplied with ABA derivatives substituted in the 3-position or with indole derivatives, the expected TV analogs can be obtained in several cases.

### Heterologous expression of XtvAB from *X*. *indica*

According to *Xenorhabdus* genome analysis (Fig 1), *X*. *eapokensis* strain DL20 not only encodes the NRPS of the TV BGC but also four genes of the *aroX* operon, encoding genes for TV precursor biosynthesis (S2 Table). Additionally, the production of **1**–**5** in the wildtype strain suggested the presence of different anthranilic acid precursors in the producer. In contrast, cultivation of *X*. *indica* did not show production of **1** or derivatives thereof. Genetic analysis revealed the presence of the TV NRPS in *X*. *indica* but absence of the *aroX* operon in proximity to the TV NRPS as shown for *K*. *oxytoca* [28] and some *Xenorhabdus* strains (Fig 1). This raised the question whether the *X*. *indica* TV NRPS is in principle functional, if sufficient supply of TV biosynthetic precursors is available. To test the biosynthetic capacity of *X*. *indica*, the corresponding NRPS (XtvAB, both proteins show 73% pairwise amino acid identity to the *X*. *eapokensis* homolog) encoding genes were heterologously expressed in *E*. *coli*.

### *E*. *coli* TV production and mutasynthesis

**Fig 5:**
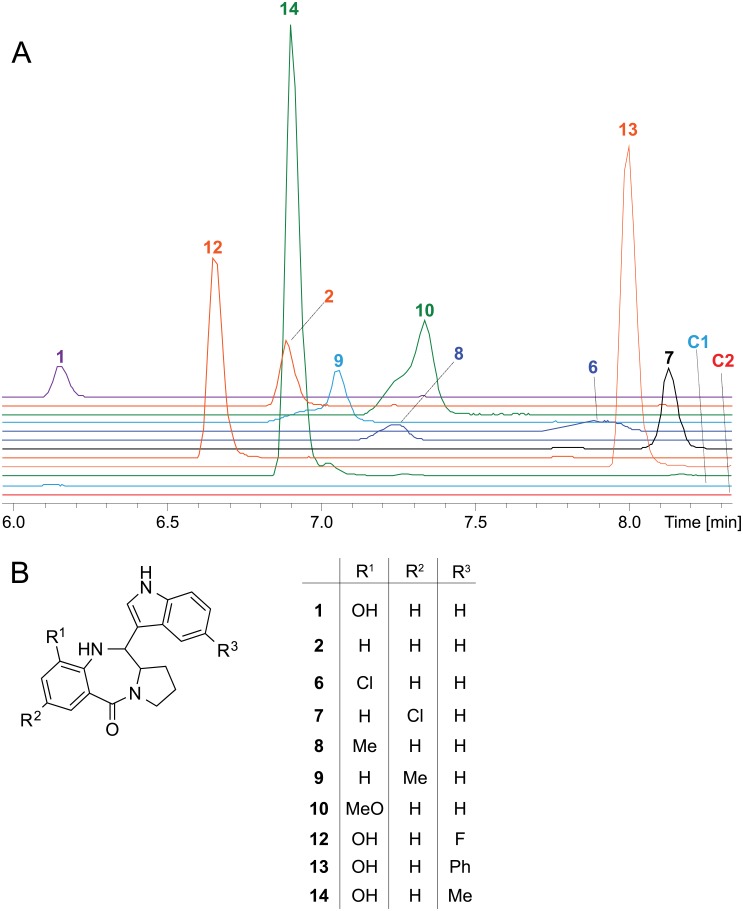
UPLC-HRMS/MS analysis of a crude extract of induced *E*. *coli* DH10B *entD*::*mtaA* carrying pHW10 (containing *xtvAB* from *X*. *indica*) grown in LB medium supplemented with different ABAs according to S3 Table. (A) Extracted ion chromatograms of TV (**1**), 9-deoxy TV (**2**) and TV analogs (**6**–**10** and **12**–**14**) with substitution patterns listed in (B). Identified ions and predicted sum formulas are listed in (Table 1), MS/MS data is shown in (Fig 4 and S3 Fig). Control C1 (non-induced *E*. *coli* DH10B *entD*::*mtaA* pHW10 with 1 mM 3-hydroxy anthranilic acid) and C2 (induced *E*. *coli* DH10B *entD*::*mtaA* pHW10 without supplementation).

Cultivation of *E*. *coli* DH10B *entD*::*mtaA* including the arabinose inducible tilivalline production vector in LB media supplemented with 3-HAA led to a TV production in the range of 0.016 mg/L in LB broth (Fig 5) thus *xtvAB* of *X*. *indica* is indeed functional but production rate is much lower compared to the *X*. *eapokensis* wildtype (Fig 2). If arabinose was lacking (Fig 5C1) or no 3-HAA was added (Fig 5C2), no production of **1** was observed. Mutasynthesis experiments as performed in *X*. *eapokensis* (S3 Table) led to the production of TV analogs **2**, **6**–**10** and **12**–**14** as confirmed by MS/MS analysis (S3 Fig). Similar to the *X*. *eapokensis* experiment 5-methoxy ABA was also not accepted as substrate. In contrast, the TV analog **9** (from incorporation of 5-methyl ABA) was observed but not TV analog **11** and **15**. However, these results in general show the use of *E*. *coli* as expression host as another possibility to obtain TV analogs.

## Discussion

Following the first observation of tilivalline (**1**) production in entomopathogenic *Xenorhabdus* bacteria [33], this study examines in detail the production of TV and analogs thereof using UPLC-MS/MS techniques in combination with *in silico* genome mining strategies.

*X*. *eapokensis* and seven more *Xenorhabdus* strains harbor the TV BGC with similarities to the TV BGC described for the human gut pathobiont *K*. *oxytoca* [28]. Following *in silico* analyses, two different TV BGC architectures were found. Both contain a bimodular NRPS, similar to BGCs of structural pyrrolobenzodiazepine relatives anthramycin [16–18], sibiromycin [14,15] and tomaymycin [19–21,38]. Furthermore the TV BGC includes the *aroX* operon, harboring several genes important for TV precursor synthesis as proposed in a TV biosynthesis pathway for *K*. *oxytoca* [34,36].

**Fig 6:**
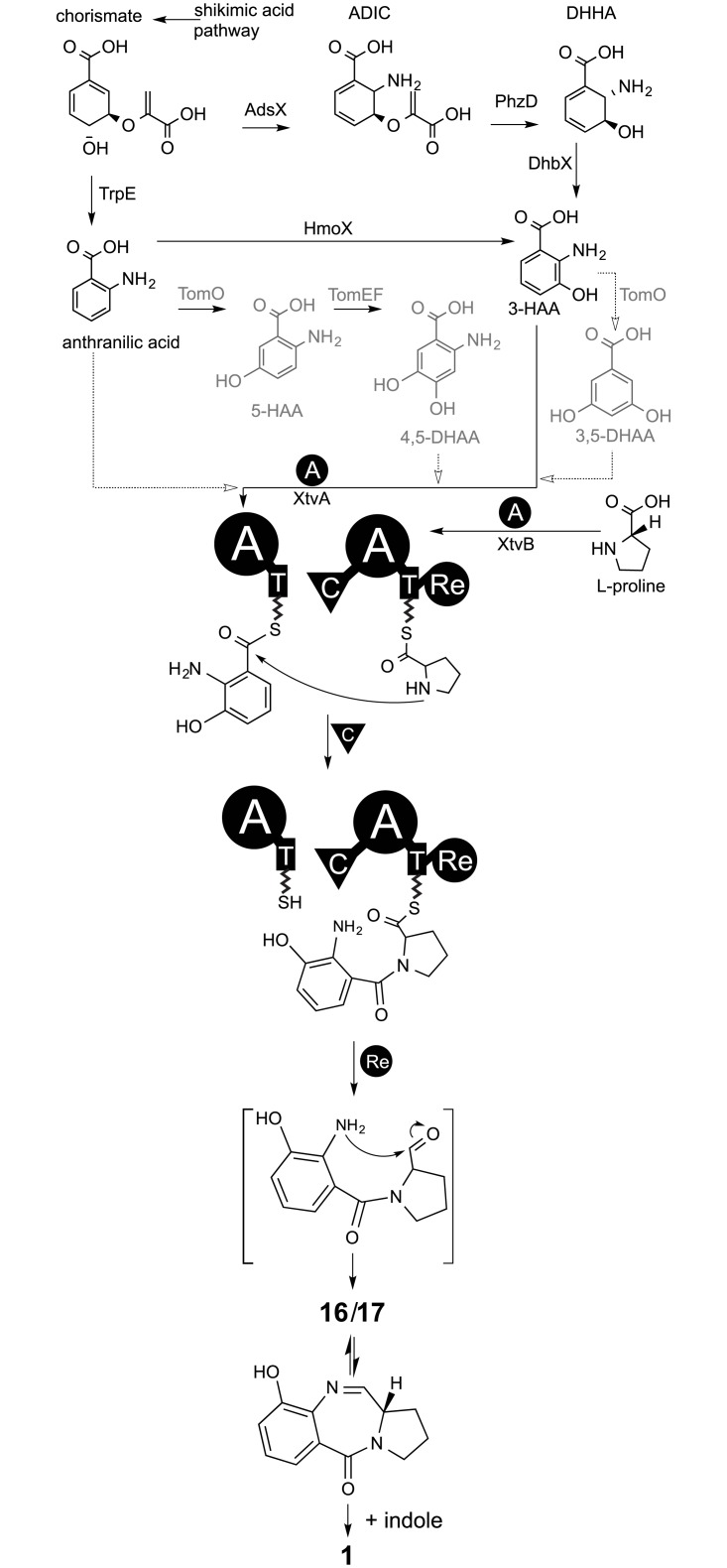
Proposed tilivallin (1) biosynthesis of *X*. *eapokensis*. Chorismate is a main product of the shikimic acid pathway modified by various enzymes to precursors accepted by XtvA. NRPS enzymes XtvA and XtvB activate anthranilic acid or proline (and analogs thereof), respectively, due to ATP hydrolysis and bind resulting aminoacyl adenylates to corresponding thiolation domains (T). After condensation domain (C) mediated reaction of the T domain bound aminoacyls, resulting dipeptide undergoes a reductive cyclisation by action of the terminal reduction domain (Re) release mechanism resulting a pyrrolobenzodiazepine core structure (**16**, **17**). Finally post NRPS, free indole (or derivatives thereof) is added non enzymatically, resulting in the production of **1**. Compound abbreviations: ADIC = 2-amino-4-deoxy isochorismate, DHHA = 6-amino-5-hydroxycyclohexa-1,3-diene-1-carboxylate, 3-HAA = 3-hydroxyanthranilic acid, 5-HAA = 5-hydroxyanthranilic acid, 3,5-DHAA = 3,5-dihydroxyanthranilic acid, 4,5-DHAA = 4,5-dihydroxyanthranilic acid. Enzyme abbreviations: AdsX = ADIC synthase, PhzD = ADIC hydrolase, DhbX = DHHA dehydrogenase, TomO = salicylyl-coenzyme A 5-hydroxylase (anthranlilic acid oxygenase), TomEF = phenol-2-monooxygenase reductase/oxygenase, XtvAB = TV NRPS module 1 and 2.

Following these proposals and genetic analyses this study elucidates the biosynthesis pathway for *Xenorhabdus* (Fig 6). Production of **2** is most likely explained by XtvA incorporation of anthranilic acid, a conversion product of chorismate by the anthranilate synthase homolog TrpE, originally being part of the tryptophan biosynthesis (S2 Table). An alternative anthranilate production pathway due to tryptophan catabolism via kynurenine seems unlikely, due to the lack of the key enzymes KynABU not encoded in *Xenorhabdus* genomes (data not shown). Chorismate in turn is a main product of the shikimic acid pathway. Homologs of all shikimic acid pathway enzymes are present in *X*. *eapokensis* with amino acid sequence identities >50% to referred query sequences (S2 Table). Presence of **1** with its typical hydroxylation at position 9 requires 3-HAA as XtvA substrate [28,34]. In general, there are two possible pathways to 3-HAA: First, action of AdsX, PhzD (or the *Klebsiella* homolog IcmX, respectively) and DhbX. All three enzymes are encoded in the *aroX* operon in *Klebsiella* and *X*. *eapokensis* (amino acid sequence similarities >48%) (Fig 1, S2 Table). The 2-amino-2-deoxy-isochorismate synthase AdsX converts chorismate to 2-amino-4-deoxy isochorismate (ADIC), while the ADIC hydrolase PhzD and the DHHA dehydrogenase DhbX in turn hydrolyze and dehydrate ADIC to 6-amino-5-hydroxycyclohexa-1,3-diene-1-carboxylate (DHHA) and finally to 3-HAA (Fig 6). Previous work [36] has demonstrated that *Klebsiella oxytoca* AHC-6 Δ*adsX*, Δ*icmX* and Δ*dhbX* deletion mutants are all impaired in TV production. Contrary, in *K*. *oxytoca* strain MH43-1 mutants in the *icmX* homolog *phzD* were still producing **1** [34]. Another proposed pathway to 3-HAA is via the anthranilate-3-monooxygenase HmoX but deletion of the corresponding gene in *K*. *oxytoca* MH43-1 and AHC-6 did not impair the production of **1** [34,36].

Furthermore, presence of **3** and **5** suggest a dihydroxylated ABA as additional XtvA substrate. Enzymatic conversion from anthranilic acid to 4,5-dihydroxy anthranilic acid (4,5-DHAA) is described in tomaymycin synthesis [21,38] by the salicylyl-CoA-5-hydroxylase TomO and the phenol-2-monooxygenase reductase/oxygenase TomEF (Fig 6) but resulting **3** and **5** would lack the expected 9-hydroxylation. So, it is proposed that 3-HAA serves as an alternative substrate for TomO mediated 5-hydroxylation, resulting in 3,5-dihydroxy anthranilic acid (3,5-DHAA) but the pathway leading to its production has not been fully elucidated yet. Interestingly, occurrence of TV derivatives **4** and **5** suggest a desaturation of the PBD C-ring (S2 Fig) that could either occur prior or after NRPS catalyzed incorporation. Characterized proline dehydrogenases PutA [39] and DpdH [40], both involved in proline degradation to glutamate, produce 1-pyrroline-5-carboxylic acid and 1-pyrroline-2-carboxylic acid, respectively. Additionally, LhpD, a cis-3-hydroxy-L-proline dehydratase [41] is proposed to produce 1-pyrroline-2-carboxylic acid. However, both mentioned pyrroline carboxylic acids are unlikely the XtvB substrate for C domain catalyzed reaction with XtvA because T domain attached 1-pyrroline-5-carboxylic acid or 1-pyrroline-2-carboxylic acid do not provide a free nitrogen atom, required for the C domain mediated nucleophilic attack to form a peptide bond. Another possibility are NRPS *in trans* tailoring enzymes as shown for Oxy proteins in teicoplanin biosynthesis [42].

Phylogenetic studies on the TV NRPS of *K*. *oxytoca* (NpsAB) addressing A domain substrate adenylation specificity suggest, that NpsA accepts anthranilic acid derivatives and NpsB pyrrole containing molecules, forming the TV precursor [24,28]. This is in line with our experimental results of a precursor directed biosynthesis approach of *X*. *eapokensis* (Fig 2) and mutasynthesis studies of an XtvAB expressing *E*. *coli* strain (Fig 5), where TV is only built in the presence of supplemented 3-hydroxy anthranilic acid.

During NRPS reaction, both T domain bound aminoacyls are fused by the C domain and subsequent action of the terminal reductase domain releases a dipeptidyl aldehyde that undergoes intramolecular cyclization, resulting the PBD imine/carbinolamine intermediates **16** and **17**, latter one described as tilimycin/kleboxymycin (Table 1) [24,34,36]. Finally, **1** results from indole attacking non-enzymatically at the C11 position [24,34].

XtvAB expressing *E*. *coli* or wildtype *X*. *eapokensis* could serve as a starting point for biotechnologically produced PBDs with varying substitution in PBD A-ring, as demonstrated for tomaymycin [21] and sibiromycin [37,43]. Furthermore, enzyme catalyzed PBD synthesis is stereoselective and avoids the use of toxic chemicals in contrast to chemical PBD synthesis [44]. Substituted PBDs for their part, can serve as monomeric [45] or dimeric [46] building blocks for antibody payloads in antineoplastic chemotherapeutics due to their cytotoxic properties. For instance, anthramycin shows sequence selective DNA minor groove binding and its active imine builds an aminal linkage to guanine DNA bases [47] thereby disturbing protein-DNA interactions. Albeit TVs exact mode of action is unknown, gut epithelial cells are affected by TV *in vitro* [28]. Additionally, it has been demonstrated that tilimycin (**16**) exhibit a greater cytotoxicity than tilivalline (**1**) on epithelial Hep-2 cells [34].

Based on this bioactivity data it can be speculated that TV derivatives are also beneficial for *Xenorhabdus* in its symbiotic lifestyle. *Xenorhabdus* live in symbiosis with *Steinernema* nematodes, hunting and killing insect larvae as food source and reproductive space [1–3]. This specific lifestyle accommodates various difficulties e.g. bypassing the insect immune system, insect killing and lysis as well protecting of the insect corpse against food competitors like other soil-living microorganisms. To cope that challenges, *Xenorhabdus* produce a wide variety of natural products [5–7,48].

However, a crucial step before reproduction in the insect corpse is the infection of the prey insect. Nematodes carrying the entomopathogenic bacteria penetrate the insect through natural openings such as the mouth, anus or spiracles [49]. Having entered the insect, the nematode regurgitates the bacteria [50]. To enter deeper tissue layers or infect the insect hemocoel, the bacteria must overcome epithelial cell layers that form a barrier between tissue and environment. Hitherto no natural compounds are reported to facilitate this step of nematode/bacteria insect prey colonization so cytotoxic TV (**1**) and tilimycin (**16**) could play a key role in this early step of *Xenorhabdus/Steinernema* reproductive life cycle. Therefore, future work will address the biochemical identification of *Xenorhabdus* produced PBDs cell target(s) in an ecological context to provide new insights in bacterial nematode symbiosis system.

## Material and methods

### Identification of the TV BGC in *Xenorhabdus*

The TV producing BGC was identified using antiSMASH v3.0 [8] analyses of genomes described in (Table 1). Protein similarity calculations were performed using Geneious v6.0 and the clustalW pairwise alignment algorithm. Homologous gene search was done using algorithms of the online BLAST tool or a custom Geneious v6.0 BLAST search.

### Cultivation of microorganisms

All *E*. *coli* and *Xenorhabdus* strains were grown in liquid or on solid LB-medium (pH 7.5, 10 g/L tryptone, 5 g/L yeast extract and 10 g/L NaCl), SF-900 II SFM (Thermo Fisher Scientific) or Schneider′s Insect Medium (Sigma Aldrich). Solid media contained 1.5% (w/v) agar. *S*. *cerevisiae* strain CEN.PK 113-7D and derivatives were grown in liquid or solid YPD-medium (10 g/L yeast extract, 20 g/L peptone and 20 g/L glucose). Agar plates contained 2% (w/v) agar. Kanamycin (20 μg/mL) and G418 (200 μg/mL) were used as selection markers. All strains were cultivated at 30°C.

### Molecular biological methods

Genomic DNA of selected *Xenorhabdus* strains was isolated using the Qiagen Gentra Puregene Yeast/Bact Kit. Polymerase chain reaction (PCR) was performed with oligonucleotides obtained from Eurofins Genomics. All PCR reactions were performed using Phusion Hot Start II High-Fidelity DNA polymerase (Thermo Scientific) according to the manufacturers’ instructions. DNA purification was performed using MSB Spin PCRapace Kit (Stratec Biomedical) according to the manufacturers’ instructions. Plasmid isolation from *E*. *coli* was performed using Invisorb Spin Plasmid Mini Two Kit (Stratec Biomedical) according to the manufacturers’ instructions. Transformation of yeast cells was done according to an established protocol [51]. Successfully constructed plasmids were isolated from yeast transformants and subsequent *E*. *coli* DH10B *entD*::*mtaA* was transformed with pHW10 by electroporation.

### Construction of plasmid pHW10

The NP production plasmid pHW10 contains the TV BGC *xtvAB* from *X*. *indica* DSM 17382 [33,52]. For its construction yeast homologous recombination (ExRec) cloning was used [53]. Therefore, the shuttle vector pCX2 (genotype: 2μ ori, pBR322 ori, G418^R^, Kan^R^, pBAD promoter [54]), was linearized by *Sac*I. Next, the TV BGC was amplified by PCR using gDNA from X. *indica* using the oligonucleotides HW p037 for and HW p038 rev (5′-*GTTTCTCCATACCCGTTTTTTTGGGCTAACAGGAGGAATTCC*ATGATCACAAAACATATCGAGCA-3′, 5′-*GTCAACAGCTCCTGCAGGCGCGCCGAGCTCGAATTGCCAT*TTACTTATATTCCGGTTCATATTTTTT-3′, respectively); letters in italic indicate homologous regions of insert to the pCX2 shuttle vector. Subsequent transformation of yeast cells with linearized pCX2 and the *X*. *indica* TV BGC PCR product, isolation of pHW10 and transformation of *E*. *coli* DH10B *entD*::*mtaA* was performed as described above.

### Expression of the TV BGC

For the heterologous expression *E*. *coli* DH10B *entD*::*mtaA* pHW10 and an *E*. *coli* transformant carrying the empty vector pCX2 as a negative control were used. Strains were grown overnight in LB-media containing the appropriate selection marker. These cultures were used for inoculation of 50 mL cultures to an OD_600_ of 0.1 which contained 2% Amberlite^™^ XAD-16 (Sigma Aldrich), an appropriate selection marker and 1 mM 3-hydroxy anthranilic acid. Gene expression was induced by the addition of 0.2% (v/v) L-arabinose at the time of inoculation. Incubation was carried out for 72 h at 30°C.

### Precursor directed biosynthesis of *X*. *eapokensis*

*X*. *eapokensis* was grown in liquid SF-900 II SFM (Thermo Fisher Scientific) in presence of 2% Amberlite^™^ XAD-16 (Sigmal Aldrich) or SF-900 supplemented with 1 mM of 2-amino-3-chlorobenzoic acid, 2-amino-5-chlorobenzoic acid, 2-amino-3-methylbenzoic acid, 2-amino-5-methylbenzoic acid, 2-amino-3-methoxybenzoic acid, 2-amino-5-methoxybenzoic acid, anthranilic acid, 3-hydroxy anthranilic acid, 3-methyl proline, 3-benzyl proline, or 4-hydroxy proline, pipecolic acid, and 2 mM of 5-fluoro indole, 5-phenyl indole, or 5-methyl indole and 3 mM of 7-azaindole (all chemicals were purchased from Sigma Aldrich). The cultures were cultivated at 30°C for three days with a starting OD_600_ of 0.1 and cultures were extracted as described below.

### Mutasynthesis

For mutasynthesis experiments *E*. *coli* DH10B *entD*::*mtaA* pHW10 cultures were grown at 30 °C for three days in presence of 2% Amberlite^™^ XAD-16 with a starting OD_600_ of 0.1 and the TV BGC was induced as described above. LB medium was supplemented as described before for the precursor directed biosynthesis of *X*. *eapokensis*. Cultures without supplementation of 2-amino benzoic acid analogs were additionally supplemented with 1 mM of 3-hydroxy anthranilic acid. Finally, cultures were harvested and extracted as described below.

### Preparation of culture extracts

After cultivation, the Amberlite^™^ XAD-16 was harvested and extracted by shaking using 25 mL of n-butanol for 60 min at 30 °C. The organic phase was filtered and evaporated to dryness under reduced pressure. The extract was reconstituted in 1 mL methanol and a 1:10 dilution was used for UPLC-MS analysis.

### Ultra performance liquid chromatography high-resolution mass spectrometry (UPLC-HRMS)

UPLC-ESI-HRMS/MS analysis was performed with an UltiMate 3000 system (Thermo Fisher) coupled to an Impact II qTof mass spectrometer (Bruker) with MeCN/0.1% formic acid in H_2_O (5:95 → 50:50% over 13 min followed by 50:50 → 95:5 over 2 min, flow rate 0.4 mL min^-1^). A 10 mM sodium formate solution served as an internal mass calibrant. The following MS settings were used: (i) source settings: capillary voltage 4500 V, nebulizer gas pressure (nitrogen) 3 bar, ion source temperature 200 °C, dry gas flow of 8 L min^-1^; (ii) general scan settings: ion polarity positive, mass range 80 to 550 *m/z*, spectra rate 3 Hz (MS and MS/MS); (iii) tune parameters: transfer funnel 1 RF 300 Vpp, Funnel 2 RF 300 Vpp, isCID off, hexapole RF 60 Vpp, stepping settings 20–50 keV; (iv) MS/MS settings: 8 precursor ions, threshold 1000 cts. (absolute), activated active exclusion after 3 spectra and 0.5 min release time, active precursor reconsidering factor 4, smart exclusion 2 times.

### Network analysis

Raw MS data of extracts from *X*. *eapokensis* cultured in SF-900 was exported from DataAnalysis v4.3 (Bruker) in the .mzXML file format. A molecular network was created using the MS cluster online workflow at GNPS (http://gnps.ucsd.edu/ProteoSAFe/static/gnps-splash.jsp). Network analysis parameters were set as follows: parent mass tolerance of 0.03 Da, MS/MS fragment ion tolerance of 0.05 Da. Consensus spectra that contained less than one spectra were discarded. A network was then created where edges were filtered to have a cosine score above 0.6 and more than five matched peaks. Edges between two nodes were kept in the network only if each of the nodes appeared in each other's respective top 7 most similar nodes.

Cytoscape v3.5.1 [55] was used to visually display the data as a network of nodes and edges and organized with the edge weighted force directed layout. Node annotation was manually performed. Dataset is available under MassIVE id: MSV000081778.

### Quantification of TV production

A 0.5 mg TV standard was dissolved in 1 mL methanol and dilutions were generated in a range from 1:1000 to 1:10,000 and measured in technical triplicates using UPLC-HR-MS. Signal area counts of EIC *m/z* 334.1550 [M+H]^+^ were plotted against concentration, where 1:10,000, 1:2000 and 1:1000 resulted in a linear function (R^2^ = 0.998) used for quantification. The calibration curve is shown in S5 Fig. Other TV derivatives were not quantified but are shown according to their ion intensities in the production cultures.

## Supporting information

S1 Fig UPLC-HRMS/MS analysis of a crude extract of *X*. *eapokensis* grown in LB medium. (A) Structure of tilivallin (**1**) chemical structure. (B) Extracted ion chromatogram (EIC) of **1**, *m/z* 334.1550 [M+H]^+^. MS/MS spectrum of natural (C) and synthetic **1** (D). Mass signals are annotated with predicted sum formulas, detection errors and putative molecule fragment structures. Red diamond indicates precursor ion.
(DOCX)

S2 Fig UPLC-HRMS/MS analysis of a crude extract of *X*. *eapokensis* grown in SF-900 medium. (A) MS/MS spectrum of dehydro-TV (**4**). (B) MS/MS spectrum of dehydro-dihydroxy TV (**5**). Mass signals are annotated with predicted sum formulas, detection errors and putative molecule fragment structures. Red diamond indicates precursor ion.
(DOCX)

S3 Fig UPLC-HRMS/MS analysis of a crude extract of *X*. *eapokensis* grown in SF-900 medium. Shown are MS/MS spectra of (A) 9-chloro-pyrrolobenzodiazepine (**6**), (B) 7-chloro-pyrrolobenzodiazepine (**7**), (C) 9-methyl-pyrrolobenzodiazepine (**8**), (D) 7-methyl-pyrrolobenzodiazepine (**9**), (E) 9-methoxy-pyrrolobenzodiazepine (**10**), (F) 1-methyl-9-hydroxy-pyrrolobenzodiazepine (**11**), (G) 9-hydroxy-5’-fluoro-pyrrolobenzodiazepine (**12**), (H) 9-hydroxy-5’phenyl-pyrrolobenzodiazepine (**13**), (I) 9-hydroxy-5’-methyl-pyrrolobenzodiazepine (**14**), and (J) 9-hydroxy-azaindole-pyrrolobenzodiazepine (**15**). Mass signals are annotated with predicted sum formulas, detection errors and putative molecule fragment structures. Red diamond indicates precursor ion.
(DOCX)

S4 Fig UPLC-HRMS/MS analysis of a crude extract of *X*. *eapokensis* grown in SF-900 medium. (A, B) Extracted ion chromatogram (EIC) of TV intermediates **16** and **17** with corresponding fragment mass spectra (MS/MS) (C, D).
(DOCX)

S5 Fig Calibration curve used for tilivallin quantification. Plotted are signal area counts of EIC *m/z* 334.1550 [M+H]^+^ corresponding to the used dilution series (1:10,000, 1:2,000 and 1:1,000 dilution of a 0.5 mg/mL stock soltion). Each data point represents the average area count of a triplicate measurement (SD_1:10,000_ = 653 counts, SD_1:2,000_ = 316 counts, SD_1:1,000_ = 292 counts). Graphical error indicators are below display limit. Regression equation and coefficient of determination are given in the diagram.
(DOCX)

S1 Table Bacterial strains used and analyzed in this work. (DOCX)

S2 Table Homology of proteins involved in tilivallin biosynthesis based on BLAST analysis of corresponding protein queries. “*” indicates no NCBI accession number available. “-”indicates no homolog was detected by pBLAST search.
(DOCX)

S3 Table Overview of building blocks for the production of TV derivatives in *X*. *eapokensis* precursor directed biosynthesis and *E*. *coli* DH10B *entD*::*mtaA* pHW10 mutasynthesis experiments. (DOCX)
