# Supplementary figures and images for: The benzodiazepine-like natural product tilivalline is produced by the entomopathogenic bacterium Xenorhabdus eapokensis
Source: PLoS One. 2018 Mar 29;13(3):e0194297. doi: 10.1371/journal.pone.0194297 (PMC5875774; doi:10.1371/journal.pone.0194297)

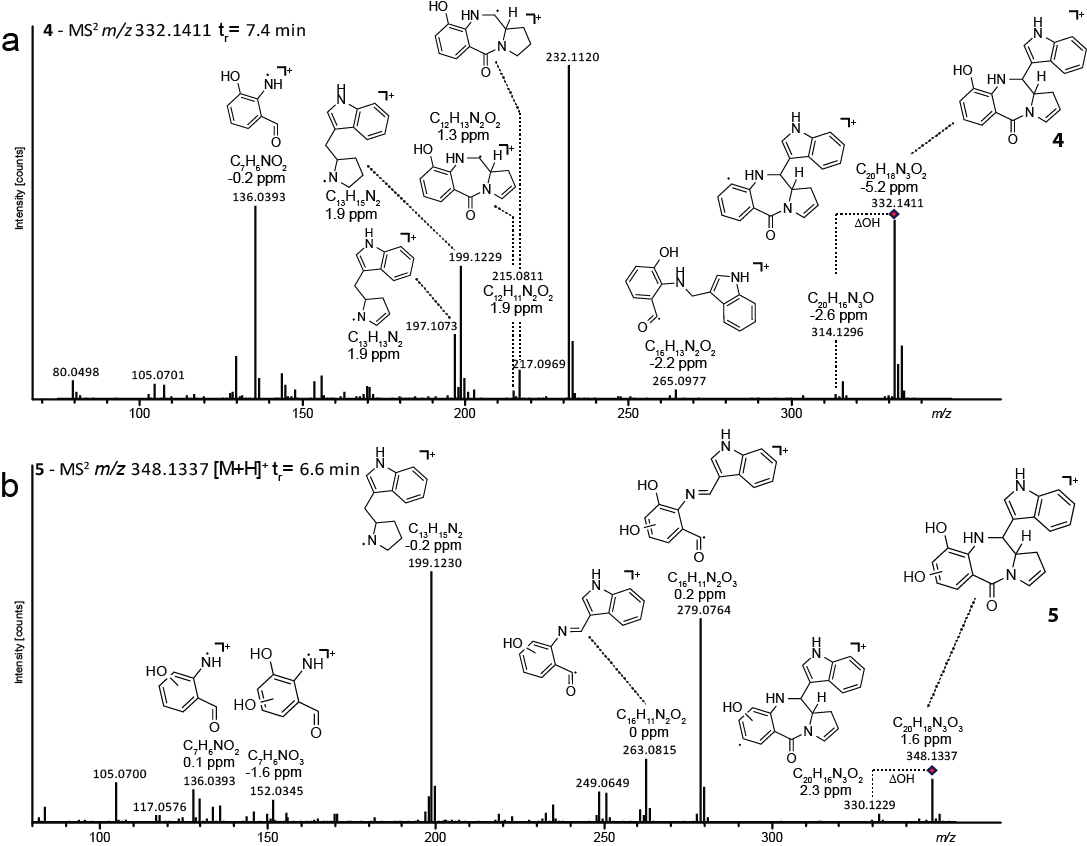

Supplement: S2 Fig — (A) MS/MS spectrum of dehydro-TV (4). (B) MS/MS spectrum of dehydro-dihydroxy TV (5). Mass signals are annotated with predicted sum formulas, detection errors and putative molecule fragment structures. Red diamond indicates precursor ion. (DOCX) [file pone.0194297.s002.docx]

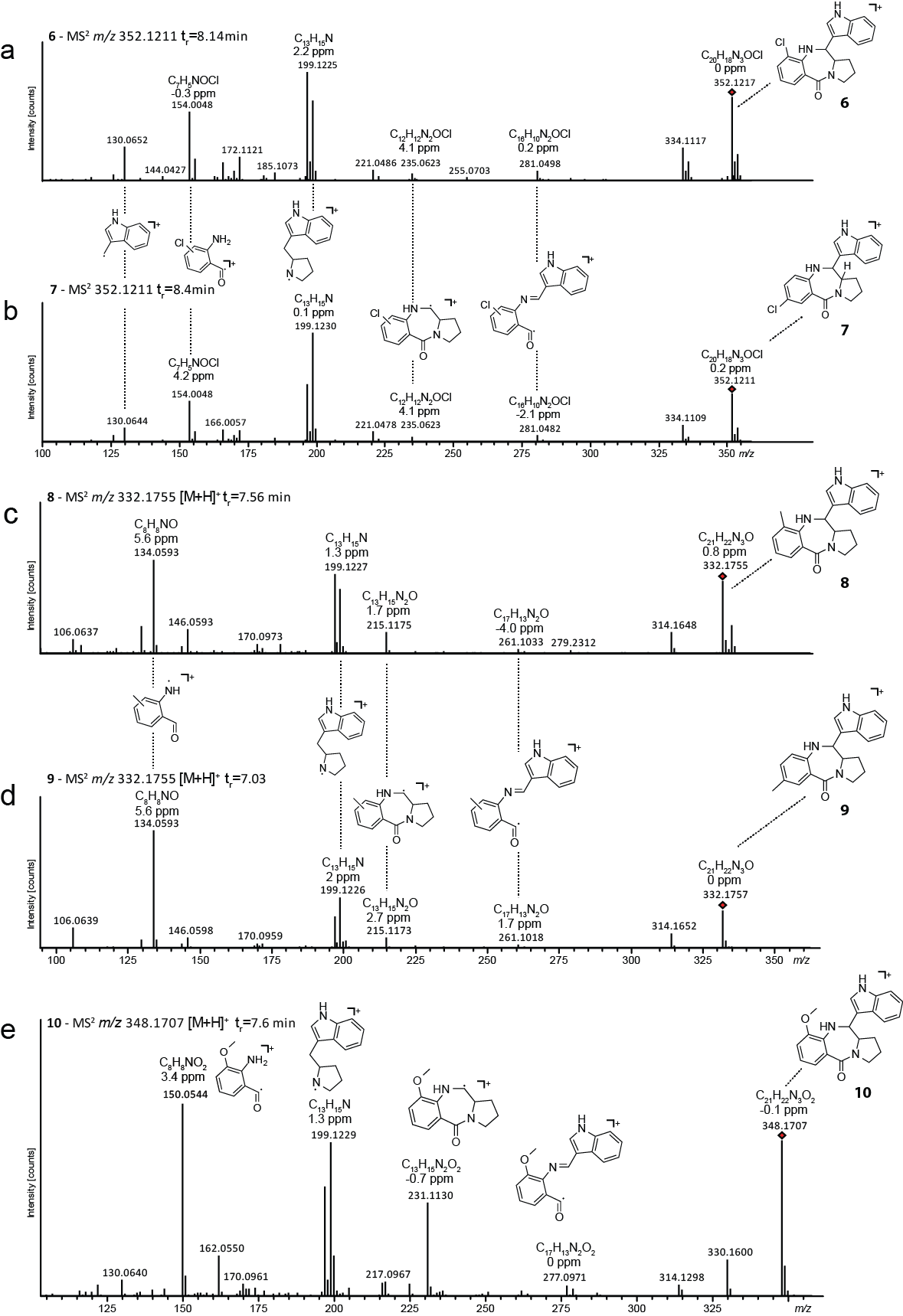


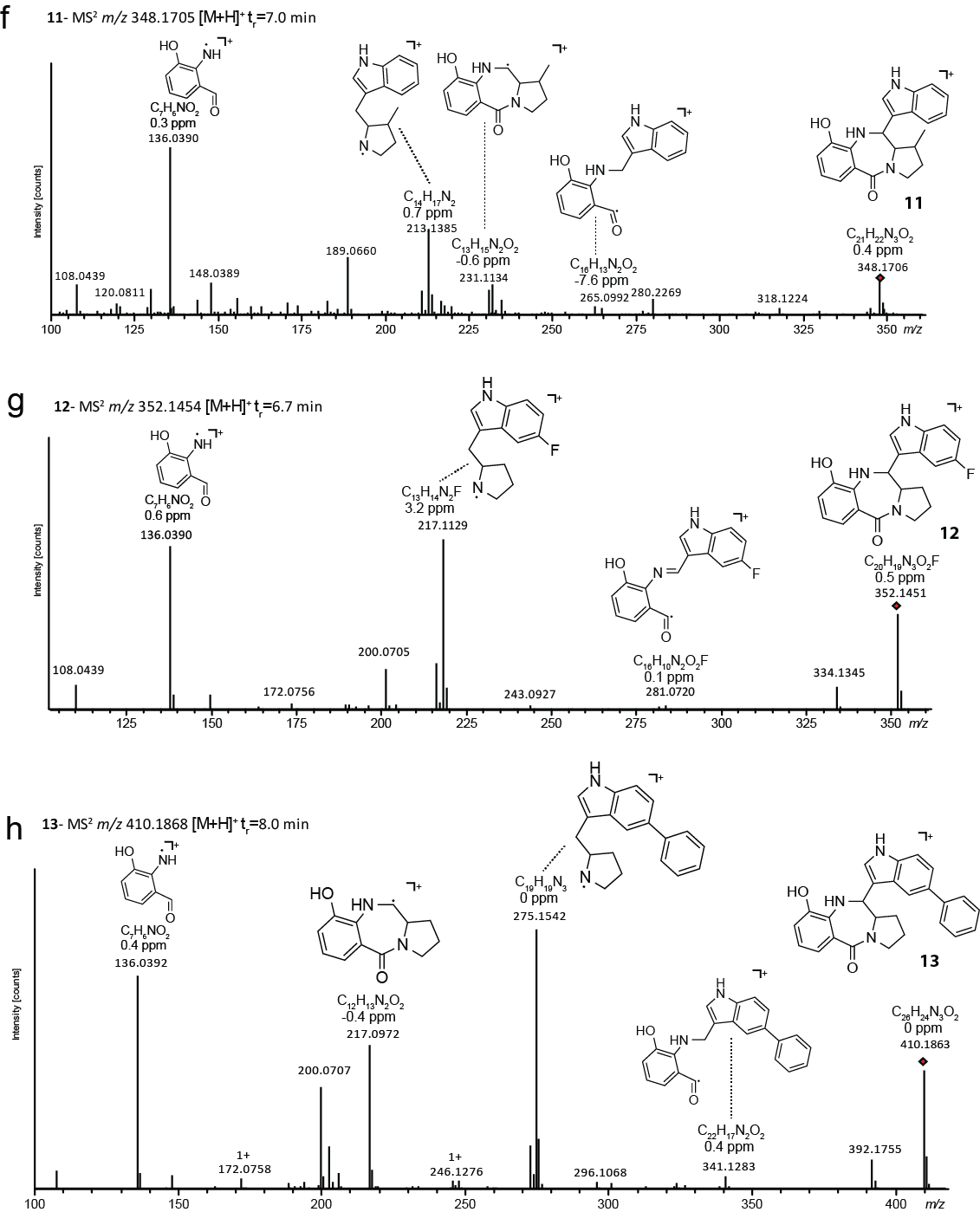


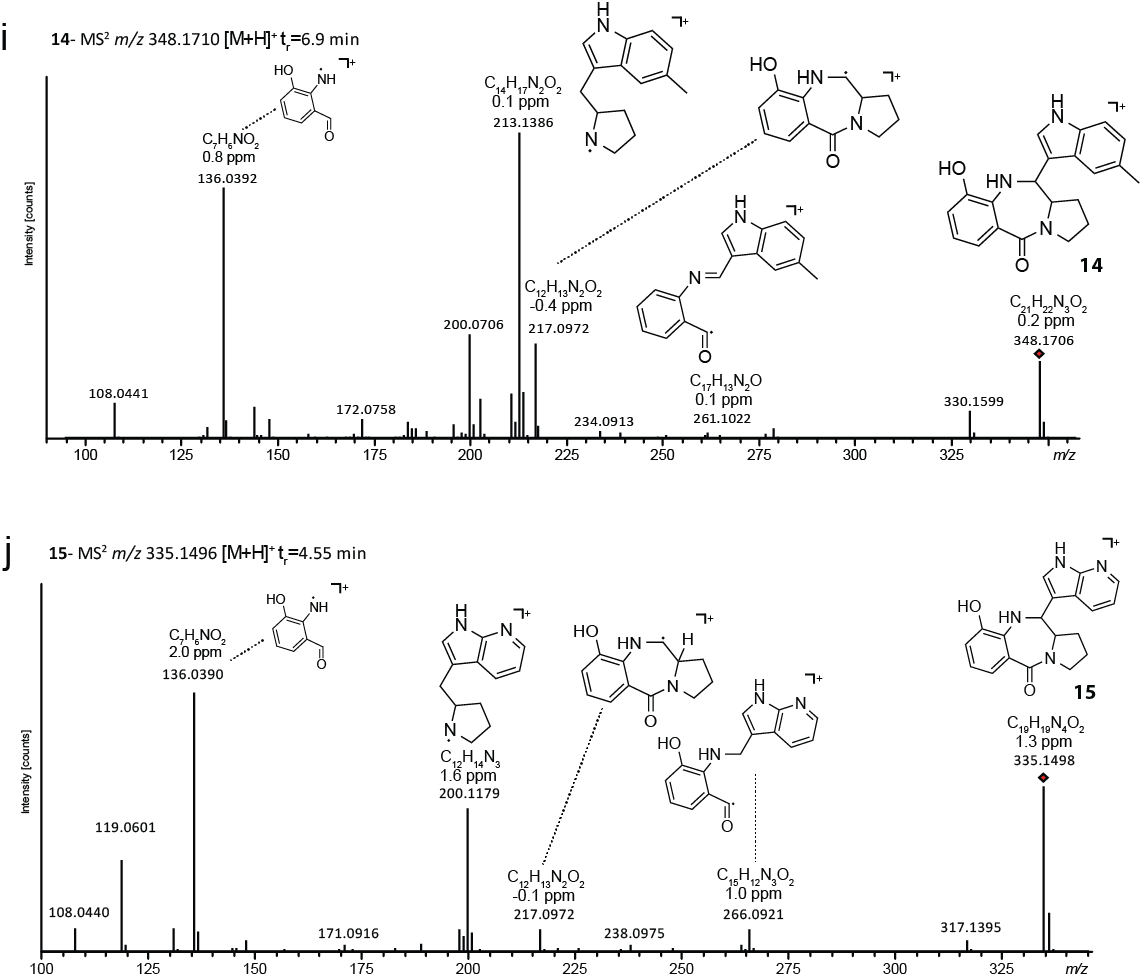

Supplement: S3 Fig — Shown are MS/MS spectra of (A) 9-chloro-pyrrolobenzodiazepine (6), (B) 7-chloro-pyrrolobenzodiazepine (7), (C) 9-methyl-pyrrolobenzodiazepine (8), (D) 7-methyl-pyrrolobenzodiazepine (9), (E) 9-methoxy-pyrrolobenzodiazepine (10), (F) 1-methyl-9-hydroxy-pyrrolobenzodiazepine (11), (G) 9-hydroxy-5’-fluoro-pyrrolobenzodiazepine (12), (H) 9-hydroxy-5’phenyl-pyrrolobenzodiazepine (13), (I) 9-hydroxy-5’-methyl-pyrrolobenzodiazepine (14), and (J) 9-hydroxy-azaindole-pyrrolobenzodiazepine (15). Mass signals are annotated with predicted sum formulas, detection errors and putative molecule fragment structures. Red diamond indicates precursor ion. (DOCX) [file pone.0194297.s003.docx]

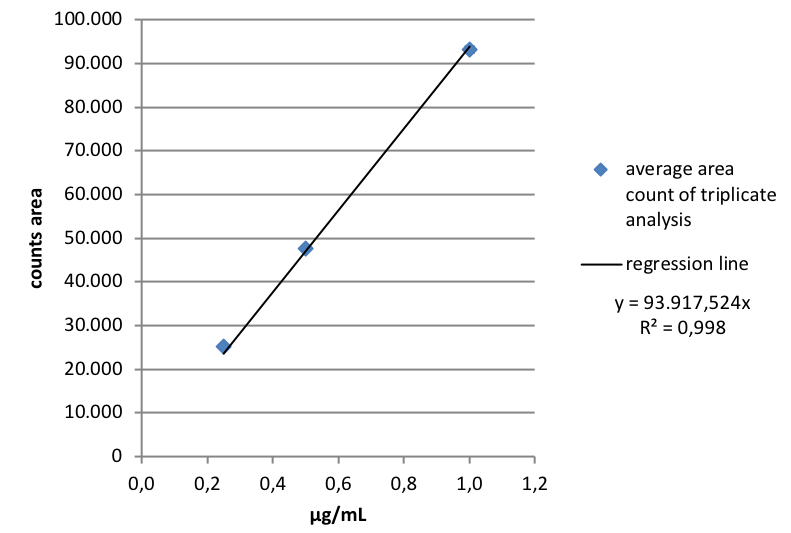

Supplement: S5 Fig — Plotted are signal area counts of EIC m/z 334.1550 [M+H]+ corresponding to the used dilution series (1:10,000, 1:2,000 and 1:1,000 dilution of a 0.5 mg/mL stock soltion). Each data point represents the average area count of a triplicate measurement (SD1:10,000 = 653 counts, SD1:2,000 = 316 counts, SD1:1,000 = 292 counts). Graphical error indicators are below display limit. Regression equation and coefficient of determination are given in the diagram. (DOCX) [file pone.0194297.s005.docx]
