# Supplementary material for: The benzodiazepine-like natural product tilivalline is produced by the entomopathogenic bacterium Xenorhabdus eapokensis
Source: PLoS One. 2018 Mar 29;13(3):e0194297. doi: 10.1371/journal.pone.0194297 (PMC5875774; doi:10.1371/journal.pone.0194297)
Supplement: S1 Table — (DOCX) [file pone.0194297.s006.docx]

| **Strain name** | **NCBI accession number** | **Genotype** | **TV BGC/ production** |
| --- | --- | --- | --- |
| *Escherichia coli* DH10B *entD::mtaA* |  | F– *mcr*A Δ(*mrr*-*hsd*RMS-*mcr*BC) Φ80*lac*ZΔM15 Δ*lac*X74 *rec*A1 *end*A1 *ara*D139 Δ(ara leu) 7697 *gal*U *gal*K *rps*L *nup*G λ– *mta*AΔ*ent*D | no/no |
| *Klebsiella michiganensis* 10-5250 | AGDP01000003.1 | wild type | yes/unknown |
| *Klebsiella oxytoca* AHC-6 | HG425356 | wild type | yes/yes |
| *Saccharomyces cerevisiae* CEN.PK 113-7D |  | MATα, MAL2-8c, SUC2 | no/no |
| *Xenorhabdus beddingii* DSM 4764 | MUBK00000000 | wild type | yes/no |
| *Xenorhabdus bovienii* SS-2004 | FN667741 | wild type | no/no |
| *Xenorhabdus budapestensis* DSM 16342 | NIBS00000000 | wild type | no/no |
| *Xenorhabdus cabanillasii* JSM26 | NJGH00000000 | wild type | yes/no |
| *Xenorhabdus doucetiae* FRM | FO704550.1 | wild type | no/no |
| *Xenorhabdus eapokensis* DL20 | [NZ_MKGQ00000000.1](https://www.ncbi.nlm.nih.gov/nuccore/1122340469) | wild type | yes/yes |
| *Xenorhabdus ehlersii* DSM 16337 | NIBT00000000 | wild type | no/no |
| *Xenorhabdus hominickii* DSM 17903 | NJAI00000000 | wild type | yes/no |
| *Xenorhabdus hominickii* strain ANU1 | GCF_001721185.1 | wild type | yes/no |
| *Xenorhabdus indica* DSM 17382 | NKHP00000000 | wild type | yes/no |
| *Xenorhabdus innexi* DSM 16336 | NIBU00000000 | wild type | no/no |
| *Xenorhabdus ishibashii* DSM 22670 | NJAK00000000 | wild type | no/no |
| *Xenorhabdus* KJ12.1 | NJCW00000000 | wild type | no/no |
| *Xenorhabdus* KK7.4 | NJAH00000000 | wild type | no/no |
| *Xenorhabdus kozodoi* DSM 17907 | NJCX00000000 | wild type | yes/no |
| *Xenorhabdus mauleonii* DSM 17908 | NITY00000000 | wild type | no/no |
| *Xenorhabdus miraniensis* DSM 17902 | NITZ00000000 | wild type | no/no |
| *Xenorhabdus nematophila* ATCC 19061 | GCF_000252955.1 | wild type | no/no |
| *Xenorhabdus nematophila* strain F1 | GCF_000389595.1 | wild type | yes/no |
| *Xenorhabdus* PB62.4 | NKHR00000000 | wild type | no/no |
| *Xenorhabdus poinarii* G6 | FO704551.1 | wild type | no/no |
| *Xenorhabdus stockiae* DSM 17904 | NJAJ00000000 | wild type | no/no |
| *Xenorhabdus szentirmaii* DSM 16338 | NIBV00000000 | wild type | no/no |
| *Xenorhabdus szentirmaii* US | NIUA00000000 | wild type | no/no |
| *Xenorhabdus* TS4 | NKHQ00000000 | wild type | no/no |
| *Xenorhabdus vietnamensis* DSM 22392 | MUBJ00000000 | wild type | no/no |
