# Supplementary material for: The benzodiazepine-like natural product tilivalline is produced by the entomopathogenic bacterium Xenorhabdus eapokensis
Source: PLoS One. 2018 Mar 29;13(3):e0194297. doi: 10.1371/journal.pone.0194297 (PMC5875774; doi:10.1371/journal.pone.0194297)
Supplement: S2 Table — “*” indicates no NCBI accession number available. “-”indicates no homolog was detected by pBLAST search. (DOCX) [file pone.0194297.s007.docx]

|  |  |  |  | | NCBI accession number of Xenorhabdus homolog (% aa identity, E-value) | | | | | | |
| --- | --- | --- | --- | --- | --- | --- | --- | --- | --- | --- | --- |
|  | function | size (aa) | *X. eapokensis*  strain DL20 | *X. indica* DSM 17382 | | *X. hominickii* DSM 17903 | *X. hominickii* AU1 | *X. kozodoi* | *X. cabanillasii* | *X. beddingii* | *X. nematophila* F1 |
| AdsX  (*K. oxytoca*) | 2-amino-4-deoxychorismate synthase | 669 | WP_074023631.1  (54, 0) | * (27, 2e^-9^) | | WP_069316706.1  (53, 0) | *  (53, 0) | WP_099141461.1  (54, 0) | WP_038259548.1  (38, 0) | WP_086110942.1  (54, 0) | WP_010848000.1  (54, 0) |
| AroA  (*K. oxytoca*) | 5-enolpyruvylshikimat-3-phosphat synthase | 428 | WP_074022514.1  (77, 0) | * (79, 0) | | WP_069316386.1  (77, 0) | *  (77, 0) | WP_099140626.1  (76, 0) | WP_069316386.1  (79,0) | WP_086111271.1  (76, 0) | CCW29539.1  (78, 0) |
| AroB  (*K. oxytoca*) | 3-dehydroquinate synthase | 365 | WP_074023339.1  (73, 0) | * (73, 1e^-169^) | | WP_069317493.1  (74, 0) | *  (74, 5e^-171^) | WP_099141335.1  (74, 0) | WP_038260025.1  (72, 0) | WP_086112193.1  (74, 0) | CCW30323.1  (75, 0) |
| AroC  (*K. oxytoca*) | chorismate synthase | 361 | WP_074023092.1  (85, 0) | * (86, 0) | | WP_069315140.1  (85, 0) | *  (85, 0) | WP_099141722.1  (86, 0) | WP_038266914.1  (86, 0) | WP_086112266.1  (86, 0) | CCW29634.1  (85, 0) |
| AroD  (*K. oxytoca*) | 3-dehydroquinate dehydratase | 150 | OKP00106.1  (77, 1e^-70^) | * (77, 2e^-72^) | | WP_069317770.1  (77, 1e^-70^) | *  (76, 1^e-62^) | WP_099142739.1  (77, 5e^-70^) | WP_038262325.1  (78, 1e^-81^) | WP_086113211.1  (77, 3e^-70^) | CCW30786.1  (77, 3e^-71^) |
| AroE  (*K. oxytoca*) | shikimate dehydrogenase | 274 | WP_074024761.1  (59, 3e^-118^) | * (56, 5e^-103^) | | WP_069318514.1  (60, 7e^-120^) | *  (60, 4e^-107^) | WP_099140451.1  (60, 2e^-122^) | WP_038269654.1  (56, 5e^-113^) | WP_086113922.1  (61, 4e^-121^) | - |
| AroF  (*E. coli)* | Tyr feedback regulated 2-keto-3-deoxy-D-arbinoheptulosonate phosphate synthase  (DAHP synthase) | 364 | WP_074023655.1  (74, 0) | * (73, 2e^-179^) | | WP_069316609.1  (74, 0) | *  (74, 0) | WP_099141486.1  (73, 0) | WP_038262437.1  (73, 0) | WP_086111790.1  (73, 0) | CCW30071.1  (73, 0) |
| AroG  (*E. coli*) | Phe feedback regulated 2-keto-3-deoxy-D-arbinoheptulosonate phosphate synthase  (DAHP synthase) | 350 | WP_074022402.1  (82, 0) | * (81, 0) | | WP_069316500.1  (83, 0) | *  (88, 0) | WP_099140729.1  (81, 0) | WP_038262084.1  (82, 0) | WP_086111156.1  (83, 0) | CCW31176.1  (81, 0) |
| AroH  (*E. coli*) | Trp feedback regulated 2-keto-3-deoxy-D-arbinoheptulosonate phosphate synthase  (DAHP synthase) | 348 | WP_074022266.1  (74, 0) | * (73, 8e^-176^) | | WP_069316046.1  (74, 0) | *  (74, 6e^-169^) | WP_099142378.1  (73, 0) | WP_038259512.1  (74, 0) | WP_086112031.1  (74, 0) | CCW31176.1  (53, 4e^-140^) |
| AroK  (*K. oxytoca*) | shikimate kinase isoenzyme I | 173 | OKP03690.1  (95, 6e^-119^) | * (93, 1e^-64^) | | WP_069317494.1  (95, 1e^-118^) | *  (95, 7e^-105^) | PHM74022.1  (95, 2e^-119^) | CDL79542.1  (94, 1e^-108^) | OTA20610.1  (95, 7e^-119^) | - |
| AroL  (*K. oxytoca*) | shikimate kinase isoenzyme II | 176 | WP_074023640.1  (46, 9e^-54^) | * (51, 1e^-51^) | | WP_069316623.1  (47, 6e^-54^) | *  (47, 3e^-48^) | WP_099141471.1  (47, 5e^-56^) | WP_038263211.1  (52, 6e^-59^) | WP_086111805.1  (46, 1e^-52^) | CCW30090.1  (44, 8e^-51^) |
| AroX  (*K. oxytoca*) | 2-keto-3-deoxy-D-arbinoheptulosonate phosphate synthase  (DAHP synthase) | 389 | WP_074023628.1  (53, 2e^-148^) | - | | WP_069316703.1  (53%, 9e^-143^,) | *  (52, 10e^-108^) | WP_099141459.1  (54, 5e^-151^) | - | - | CCW30870.1  (58, 2e^-145^) |
| DhbX  (*K. oxytoca*) | 2,3-dihydro-2,3-dihydroxybenzoate dehydrogenase | 261 | WP_074023629.1  (71, 3e^-127^) | * (39, 2e^-48^) | | WP_069316704.1  (70, 2e^-125^) | *  (70, 5e^-112^) | WP_099141460.1  (73, 5e^-130^) | WP_038264814.1  (39, 1e^-55^) | WP_086110944.1  (72, 7e^-128^) | WP_010847998.1  (70, 1e^-125^) |
| HmoX  (K. oxytoca) | anthranilate 3-monooxygenase | 520 | WP_074024671.1  (53, 0) | * (53, 7e^-175^) | | WP_069318333.1  (53, 0) | *  (52, 5e^-175^) | WP_099142853.1  (53, 0) | WP_038261961.1  (53, 0) | WP_086112357.1  (53, 0) | CCW30777.1  (53, 0) |
| NpsA  (*K. oxytoca*) | TV NRPS module 1 | 608 | WP_074023627.1  (50, 1e^-162^) | * (48, 7e^-157^) | | WP_069316702.1  (69, 0) | *  (47, 8e^-152^) | WP_099141458.1  (72, 5e^-130^) | WP_051502390.1  (90, 0) | WP_086110945.1  (69, 0) | WP_010847996.1  (71, 0) |
| NpsB  (*K. oxytoca*) | TV NRPS module 2 | 1458 | WP_074023626.1  (51, 0) | * (51, 0) | | WP_069316701.1  (50, 0) | *  (50, 0) | WP_099141457.1  (51, 0) | WP_084766479.1  (51, 0) | WP_086110947.1  (52, 0) | CCW30868.1  (51, 0) |
| PhzD (IcmX homolog)  (*Pseudomonas sp.)* | 2-amino-4-deoxychorismate hydrolase | 292 | WP_074023412.1  (48, 9e^-70^) | * (45, 7e^-55^) | | WP_069317068.1  (47, 8e^-69^) | *  (47, 1e^-62^) | PHM73866.1  (59, 2e^-34^) | WP_038264811.1  (47, 6e^-68^) | WP_086113657.1  (45, 4e^-59^) | CCW30872.1  (48, 2e^-69^) |
| TomE (*Streptomyces sp.)* | phenol-2-monoxygenase, reductase component | 182 | WP_074024068.1  (35, 2e^-23^) | * (34, 1e^-20^) | | - | *  (30, 4e^-13^) | WP_099142537.1  (35, 4e^-23^) | - | - | - |
| TomF  (*Streptomyces sp.)* | phenol-2-monoxygenase, oxygenase component | 520 | WP_074024671.1  (53, 0) | * (53, 0) | | WP_069318333.1  (53, 0) | *  (54, 0) | WP_099142853.1  (53, 0) | WP_038261961.1  (53, 0) | WP_086112357.1  (53 ,0) | WP_010847910.1  (53, 0) |
| TomO (*Streptomyces sp.)* | salicylyl-coenzyme A (CoA) 5-hydroxylase | 786 | WP_074024881.1  (39, 1e^-82^) | * (40, 3e^-83^) | | WP_069317120.1  (37, 7e^-83^) | *  (37, 1e^-79^) | WP_099142589.1  (39, 2e^-82^) | - | - | - |
| TrpE  (*E. coli*) | anthranilate synthase | 519 | WP_074023527.1  (66, 0) | * (64, 0) | | WP_069315854.1  (66, 0) | *  (66, 0) | WP_099141180.1  (66, 0) | WP_038270115.1  (65, 0) | WP_086112569.1  (64, 0) | CCW32517.1  (68, 0) |
