# Supplementary material for: The benzodiazepine-like natural product tilivalline is produced by the entomopathogenic bacterium Xenorhabdus eapokensis
Source: PLoS One. 2018 Mar 29;13(3):e0194297. doi: 10.1371/journal.pone.0194297 (PMC5875774; doi:10.1371/journal.pone.0194297)
Supplement: S3 Table — (DOCX) [file pone.0194297.s008.docx]

| precursor | concentration added [mM] | production in  *X. eapokensis* | heterologous production in *E. coli* |
| --- | --- | --- | --- |
| 2-amino-3-chlorobenzoic acid | 1 | **6** | **6** |
| 2-amino-5-chlorobenzoic acid | 1 | **7** | **7** |
| 2-amino-3-methylbenzoic acid | 1 | **8** | **8** |
| 2-amino-5-methylbenzoic acid | 1 | - | **9** |
| 2-amino-3-methoxybenzoic acid | 1 | **10** | **10** |
| 2-amino-5-methoxybenzoic acid | 1 | - | - |
| anthranilic acid | 1 | **2** | **2** |
| 3-hydroxy anthranilic acid | 1 | **1** | **1** |
| 3-methyl proline | 1 | **11** | **-** |
| 3-benzyl proline | 1 | - | - |
| 4-hydroxy proline | 1 | - | - |
| pipecolic acid | 1 | - | - |
| 5-fluoro indole | 2 | **12** | **12** |
| 5-phenyl indole | 2 | **13** | **13** |
| 5-methyl indole | 2 | **14** | **14** |
| 7-azaindole | 3 | **15** | - |

“-“ = not detected
