# Supplementary material for: The benzodiazepine-like natural product tilivalline is produced by the entomopathogenic bacterium Xenorhabdus eapokensis
Source: PLoS One. 2018 Mar 29;13(3):e0194297. doi: 10.1371/journal.pone.0194297 (PMC5875774; doi:10.1371/journal.pone.0194297)
Supplement: Supplementary file 5 [file pone.0194297.s001.docx]

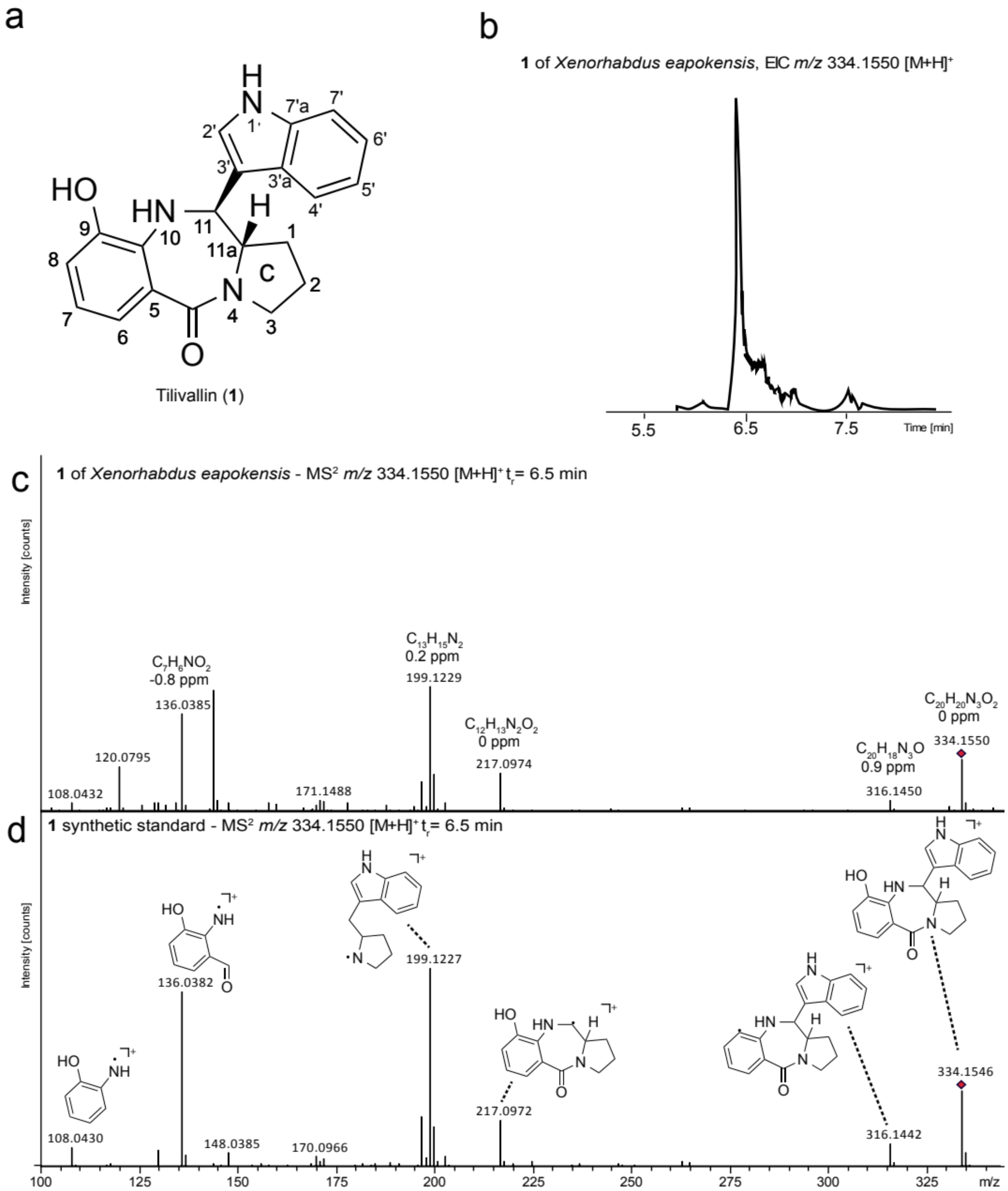
a
Tilivallin (1)
b
1 of Xenorhabdus eapokensis, EIC m/z 334.1550 [M+H]+
Time [min]
c
1 of Xenorhabdus eapokensis - MS2 m/z 334.1550 [M+H]+ tr= 6.5 min
Intensity [counts]
C7H6NO2 -0.8 ppm 136.0385
C13H15N2 0.2 ppm 199.1229
C12H13N2O2 0 ppm 217.0974
C20H18N3O 0.9 ppm 316.1450
C20H20N3O2 0 ppm 334.1550
108.0432
120.0795
171.1488
d
1 synthetic standard - MS2 m/z 334.1550 [M+H]+ tr= 6.5 min
108.0430
136.0382
148.0385
170.0966
199.1227
217.0972
316.1442
334.1546
m/z
