# Supplementary material for: The benzodiazepine-like natural product tilivalline is produced by the entomopathogenic bacterium Xenorhabdus eapokensis
Source: PLoS One. 2018 Mar 29;13(3):e0194297. doi: 10.1371/journal.pone.0194297 (PMC5875774; doi:10.1371/journal.pone.0194297)
Supplement: Supplementary file 6 [file pone.0194297.s004.docx]

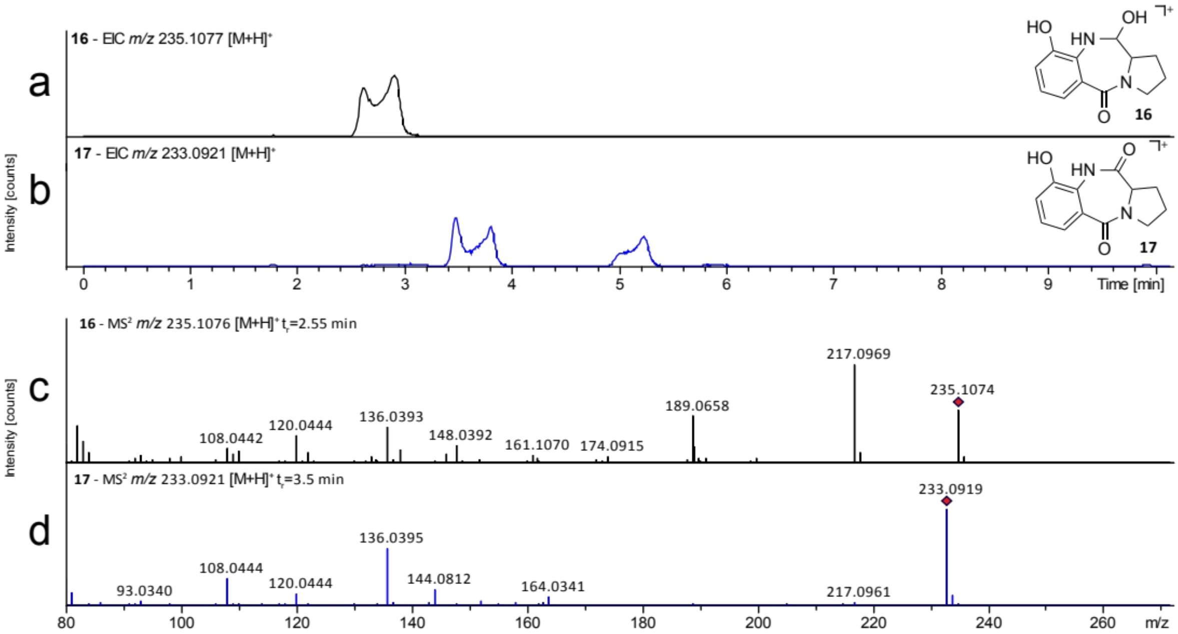
16 - EIC m/z 235.1077 [M+H]+
17 - EIC m/z 233.0921 [M+H]+
a
b
c
d
Intensity [counts]
Time [min]
16 - MS2 m/z 235.1076 [M+H]+ tr=2.55 min
17 - MS2 m/z 233.0921 [M+H]+ tr=3.5 min
217.0969
235.1074
189.0658
136.0393
120.0444
148.0392
108.0442
161.1070
174.0915
233.0919
136.0395
108.0444
144.0812
120.0444
164.0341
93.0340
217.0961
m/z
HO
HN
OH
N
O
16
17
